# Handling the Imbalanced Problem in Agri-Food Data Analysis

**DOI:** 10.3390/foods13203300

**Published:** 2024-10-17

**Authors:** Adeyemi O. Adegbenjo, Michael O. Ngadi

**Affiliations:** 1 Department of Bioresource Engineering, McGill University, 21111 Lakeshore Road, Ste-Anne-de-Bellevue, Montreal, QC H9X 3V9, Canada; 2 Process Quality Engineering, School of Engineering and Technology, Conestoga College Institute of Technology and Advanced Learning, 299 Doon Valley Drive, Kitchener, ON N2G 4M4, Canada

**Keywords:** imbalanced data, food processing, machine learning algorithms, innovation adoptability

## Abstract

Imbalanced data situations exist in most fields of endeavor. The problem has been identified as a major bottleneck in machine learning/data mining and is becoming a serious issue of concern in food processing applications. Inappropriate analysis of agricultural and food processing data was identified as limiting the robustness of predictive models built from agri-food applications. As a result of rare cases occurring infrequently, classification rules that detect small groups are scarce, so samples belonging to small classes are largely misclassified. Most existing machine learning algorithms including the K-means, decision trees, and support vector machines (SVMs) are not optimal in handling imbalanced data. Consequently, models developed from the analysis of such data are very prone to rejection and non-adoptability in real industrial and commercial settings. This paper showcases the reality of the imbalanced data problem in agri-food applications and therefore proposes some state-of-the-art artificial intelligence algorithm approaches for handling the problem using methods including data resampling, one-class learning, ensemble methods, feature selection, and deep learning techniques. This paper further evaluates existing and newer metrics that are well suited for handling imbalanced data. Rightly analyzing imbalanced data from food processing application research works will improve the accuracy of results and model developments. This will consequently enhance the acceptability and adoptability of innovations/inventions.

## 1. Introduction

A dataset is said to be imbalanced if the classification groups in the data are not equally represented; in other words, if the classification data tend to have skewed class proportions [1,2]. The specific group with very few training examples is usually called the rare (minority or positive) class, while the other with many examples is called the prevalent (majority or negative) class. Imbalanced data situations exist in most fields of endeavor, like the biomedical, surveillance, and security industries, insurance, management/finance [3,4], and the agri-food sectors. Since rare cases occur infrequently, classification rules that detect small groups tend to be scarce and samples belonging to small classes are largely misclassified compared to those of prevalent classes [5]. It is therefore of great necessity that emerging research studies in food and agricultural applications pay close attention to addressing the menace of imbalanced data distribution, which has long been neglected in food and agricultural data analyses.

In real-world situations, for example, in the study of chicken egg fertility/early embryonic development detection, the occurrence of fertile eggs is much more frequent than that of the non-fertile eggs in any available egg set. Indeed, it is commonly observed in industrial settings and commercial hatcheries that only up to 10% non-fertile eggs exist in any whole egg set batch. This occurrence, like in other agri-food applications, has caused major setbacks in the classification accuracies of most existing learning algorithms. Even though various classification learning algorithms, like the backpropagation neural network, decision tree, nearest neighbor, support vector machine, Bayesian network, etc., have been successfully applied in many application domains, datasets of imbalanced distribution continue to be a critical bottleneck for most classifier learning algorithms [5,6,7].

Major areas needing careful attention in terms of building a futuristic, robust predictive model have earlier been identified as sample size, analysis/modelling techniques, and the rare class data acquisition problem [8]. Solving the sample size and analysis/modelling technique challenges can be made simple and straightforward by sacrificing the time, financial, and human resources needed to acquire large enough quality datasets and trying such data painstakingly on pools of available classification algorithms and modelling techniques. However, solving the rare class (imbalanced) data problem is somehow complicated and very critical, since omitting it would eventually render ineffective the other solutions to sample size and modelling techniques. Handling the rare class data problem therefore takes the priority among others. 

The ideal approach to handling an imbalanced dataset is to initially train the data in its true distribution. If the model performs well without overfitting, then the mission is accomplished. Otherwise, the data must be restructured in a way to necessitate adequate feature learning. A model’s robustness is critical in predictive modelling, as is its parsimony. If models are not built with few and important discriminating features but with other noisy features, the calibration model may do well but lead to non-robustness of the final model. Whereas research in other fields of endeavor have demonstrated great improvement to models built from corrected imbalanced data [9,10,11], there have not been many research efforts geared towards solving the imbalanced problem in agri-food data analysis. Rather, earlier works had built and validated models on assumed balanced data, the results of which when applied to the real-world situation are very prone to doubt [12,13]. This might indeed be the major reason for the low acceptability and adoptability of such models in real industrial settings. Understanding that rightly analyzing imbalanced data from food processing application research works will aid in building robust and parsimonious models, this present study was set up to examine the existing techniques for handling imbalanced data problems in other fields of endeavor like the computer sciences and biomedical field, with a view to benefiting from the versatility of using such techniques in the agri-food sector. 

## 2. Related Works

In the computer sciences field of endeavor, Table 1 shows the degree of imbalance and ZeroR classifier performance (ZeroR Acc) in a network intrusion detection study [11,14]. The ZeroR classifier is known to be used for baseline classification, as a benchmark for other classification methods. The baseline performance, therefore, gives information about the least obtainable accurate classifier and any finally built machine learning model must have accuracy better than the baseline performance accuracy to be useful [15]. Obtaining a ZeroR classifier accuracy of 99.9% showed that learning in the present input data space cannot be considered as an artificial intelligence task unless the data structure is mapped into a more balanced data space exposable to learning. The situation at hand is already at a near perfect accuracy and so it will be difficult for any other classification method to beat this baseline performance, which is predicting only the predominant class. Still using the network intrusion detection data, Table 2 shows a combination of classifiers modelling results in the imbalanced data input space, with obtained accuracies being less than the baseline performance accuracies and hence such models cannot be of any good utility. Upon using the Synthetic Minority Oversampling Technique (SMOTE) algorithm to tackle the imbalanced situation, modelling results in terms of the overall accuracy, F1-score, recall, and precision are shown in bold font with clear optimized outputs. Although the work did not mention the newly obtained balanced ratio upon the application of the SMOTE algorithm variations, there was a clear demonstration of the versatility of the SMOTE algorithm in addressing the class imbalance problem.

Existing cases of imbalanced data scenarios in the agri-food sector include but are not limited to crop-food disease and stress detection [23,24,25], fruit bruise detection [26,27,28,29], infectious fruit/vegetable prediction [30,31], and anomaly detection [13], which includes the chicken egg fertility classification [32,33,34]. In the work of [13], computer vision technology was used to classify double-yolk duck eggs from single-yolk duck eggs. Even though identification rates of up to 99% and 100% were reported, the data used had assumed a balanced distribution of 150 single-yolk eggs and 150 double-yolk eggs, despite the fact that double-yolk eggs are not usually prevalent as single-yolk eggs. Therefore, this was an imbalanced data situation analyzed otherwise [12]. From a chicken egg fertility classification scenario [32], modelling egg fertility data in its natural imbalanced ratio state of 1:13 achieved excellent true positive rate accuracy of up to 100% but using 25 PLS components (PCs), making the model non-parsimonious. Modelling the same imbalanced egg fertility data with fewer numbers of 5 PCs shifted recognition accuracy to be in favor of the majority class. Addressing the imbalanced problem was therefore reported as a necessity towards optimizing the use of an adequate number of PCs and at the same time building a robust chicken egg fertility classification model.

Although there have not been many research endeavors addressing the imbalanced data problem in the agri-food sector, [35] reported using the SMOTE algorithm for the classification of an imbalanced wine quality dataset and obtained improved specificity and receiver operating characteristic (ROC) accuracies for the random forest classifier: 0.32, 0.88 (prior SMOTE application) and 0.96, 0.99 (after SMOTE application), respectively. Likewise, [36] attempted to change the status quo of using balanced training data for building foodborne pathogen prediction models in agricultural water. The work used resampling combination of oversampling and SMOTE approaches and reported more accurate models with resampling methodologies in comparison to non-resampled imbalanced data.

### Experimental Chicken Egg Fertility Data

To demonstrate the occurrence of the imbalance problem and the necessity of addressing the problem in the agri-food sector, a total of 9207 white-shell eggs were acquired from a commercial hatchery. Data were collected in duplicate upon 90-degree rotation inside the egg holder. All eggs were imaged prior to incubation and after every incubation day using a near-infrared (NIR) hyperspectral imaging system. Labelling information on fertility of the eggs was obtained upon egg break-out after about 10 days of incubation. Resampling approaches of SMOTE and random undersampling (Ru), in conjunction with randomization (RAND) implementation were examined for effects on classification accuracies in terms of sensitivity (SEN), specificity (SPE), area under ROC curve (AUC), and the F1-score. The 10-fold cross-validated results obtained using the K-nearest neighbors (KNN) classifier are as shown in Table 3. The very high ZeroR performance accuracy of 95.70% for imbalanced datasets S1 and S2 showed that learning the data in the present distribution cannot be considered a machine intelligence task. Even though sensitivity accuracies of 99.5% and 99.10% were a bit higher than the ZeroR baseline performance, the specificity accuracies of 0.30% and 2.90% showed these models are only shifting accuracy to be in favor of the prevalent positive class, making the models non-robust in the long run due to the problem of overfitting. Such models in practice will not generalize but will predict both classes as the majority class. This occurrence with the chicken egg fertility situation means the model will predict all fertile eggs as fertile and simultaneously predict all non-fertile eggs as equally fertile. Re-mapping the data structure into a more balanced distribution using various implementation of the resampling algorithms, ZeroR performance accuracies were first observed to be decreased and in the range of 50% to 57.90%. The new balanced distribution therefore provided appropriate baseline performance that can be improved upon, using various machine learning algorithms. By using the KNN classifier and 10-fold cross-validation, sensitivity accuracies were improved from baseline low performances to between 90.10% and 93.10%. Likewise, specificity accuracies improved from between 0.3 and 2.90% to between 97.10 and 98.30%. The potential overfitting problem was therefore resolved by using the balanced distribution data. Notable improvement when using resampling methodologies was also observed in the obtained AUC and F1-score accuracies, having values ranging from 93.80% to 98.10%. 

Despite available evidence (from non-agri-food and agri-food data) of achieving substantive model upliftment for addressing imbalanced data, there have not been rigorous research efforts in the direction of solving the inherent imbalanced data problem in agri-food applications. The remaining part of this work is therefore dedicated to examining some of the well-known state-of-the-art approaches for handling the imbalance problem in other fields of endeavour, and discussing some important metrics for imbalanced data analyses, with a view to appropriately applying such approaches and metrics in agri-food data processing and predictive modelling. 

## 3. Methodologies for Handling the Imbalanced Problem

Published studies were gathered over the internet from two primary sources, namely Google Scholar and ScienceDirect. Keywords used for the search included “Imbalanced data + machine learning + agriculture + food analysis”. Some specific searches including “review” as a keyword were also run using data acquisition technology, such as “Imbalanced data + hyperspectral imaging + review”; “Imbalanced data + hyperspectral imaging + agriculture + food analysis”. Studies from these search runs were examined from titles and abstracts for identifying those related to predictive modelling tasks and such were downloaded and considered in this work.

For most agri-food applications, the nature of imbalance is usually that of the majority class being of uttermost recognition importance as against most other external fields in which the minority class is always of uttermost recognition importance. For example, whole (unbruised) food products are ideally more abundant than bruised food products and correctly identifying all unbruised fruits/vegetables is more beneficial and economical to food industries than misclassifying some bruised fruits. Likewise, in crop-food disease detection, correctly identifying all disease-free crop-food is more beneficial than misclassifying some diseased crop-food. However, in other fields like biomedicals, correctly identifying the rare class disease subjects is more critical than misclassifying some healthy control subjects. This difference in the class of uttermost recognition importance between the agri-food cases and cases in other sectors should be considered appropriately during analysis of agri-food imbalanced data, in relation to determining the positive and negative classes. Some of the methodologies well attested to in the literature for solving the imbalanced problem are hereby discussed, including, but not limited to, data preprocessing (resampling), feature selection, recognition-based approach, cost-sensitive learning, ensemble methods, and the deep learning architectures [2,11,19,37,38,39,40,41]. 

### 3.1. Data Preprocessing (Resampling)

Data preprocessing is otherwise known as data sampling or resampling. The approach focuses on modifying class distribution towards handling class imbalance. The technique has a major advantage of being implemented independently of any underlying classifier [42]. The main task with the approach is to preprocess training data to minimize any divergence between the classes, thereby improving the initial data distributions of the prevalent and non-prevalent class to achieve a more uniform number of occurrences in each class [40,43]. The data preprocessing approach has been widely discussed under the following categories: oversampling, undersampling, and a hybrid of oversampling and undersampling. Ref. [43] successfully used data preprocessing methods of oversampling, undersampling, and a hybrid of the two to classify weld flaws with imbalanced class datasets. Oversampling increases the number of rare class occurrences by duplicating them until they are on par with the prevalent class occurrences. This approach is advantageous in that all information from the majority class is kept intact and all the occurrences of the rare class are also fully considered. Notwithstanding, researchers have reported the likelihood of overfitting occurring with this method as existing copies of instances are usually exactly duplicated. Due to this setback, more sophisticated approaches have been proposed, among which the “Synthetic Minority Oversampling Technique” (SMOTE) has become popular [6,44,45]. The main principle behind SMOTE implementation is to create new rare class (synthetic) examples via interpolation of various non-prevalent class instances (nearest neighbors) lying together, for oversampling the calibration dataset [42]. Due to the possible challenge of overgeneralization largely related to the manner of synthetic samples generation, there exist some adaptive sampling methods, proposed to reduce overgeneralization tendencies with SMOTE implementation. These methods include the use of Borderline-SMOTE, SPIDER2, Adaptive Synthetic Sampling, and Safe-Level-SMOTE algorithms [46,47,48,49].

In undersampling, the majority class instances are reduced to a smaller set comparable to the minority class, thereby at the same time preserving all the minority class occurrences. This technique nonetheless has a drawback of losing cogent information from the majority class occurrences and thereby degrades classifier effectiveness. However, since the mode of operation with undersampling is mostly based on data cleaning techniques, some data cleaning algorithms have been proposed to uplift the results of conventional undersampling implementation. Such data cleaning algorithms include the Wilson’s edited nearest neighbor (ENN) rule, the one-sided selection (OSS), Tomek Links, the neighborhood cleaning rule, and the NearMiss-2 method [50,51,52,53,54,55,56]. Combinations of data cleaning and resampling techniques have also been reported to have the potential to reduce overlapping commonly introduced by adopting resampling methods alone, and by so doing, a best percentage of implementing both undersampling and oversampling could be ascertained [57,58]. Furthermore, some cluster-based sampling algorithms have been reported to be useful for preprocessing before implementing undersampling and/or oversampling [59,60,61,62,63,64,65]. In the same vein, the application of particle swarm optimization (PSO) or genetic algorithms (GA) for correct identification of useful examples has been shown to be very helpful with imbalanced learning [4,66] and in improving SVM cl assification accuracies [67].

### 3.2. Feature Selection

There have been some research efforts reported on using feature selection to tackle the imbalanced data problem [68,69,70,71]. Features are usually ranked independent of their relationship with other features, thereby showing the effectiveness of individual features in predicting the category of each sample [39]. Ref. [71] adopted an optimal feature weighting (OFW) algorithm to select optimized features from high dimensional and imbalanced microarray data. Likewise, [72] developed a new feature selection (FAST) algorithm based on AUC and the threshold moving technique, to tackle small-sample imbalanced datasets. Feature selection is aimed at selecting a subset of “K” features (“K” being a user-defined parameter) from an original set of “L” features, so that the feature space is optimally decreased in accordance with some evaluation criteria [15,73,74]. The selection of subsets per time to be learned are usually determined using the embedded, filter, and/or wrapper methods [75,76]. While the filter approach considers the appropriateness of the selected features, independent of the classifying algorithm, the wrapper method on the other hand requires a classifier to evaluate feature appropriateness, but also can be computationally burdensome. Whereas the filter techniques are classifier-independent, simple, and fast, they are limited due to their dark knowledge of the interaction between feature subset search and classifier [73]. This disadvantage of the filter techniques is catered for in the wrapper and embedded methods. Also, there exist multivariate filters purposely developed to overcome the filter approach limitation, and these include the information gain, correlation, and learner-based feature selection [72,73,77]. Even though feature selection has been an integral part of machine learning/data mining from inception, its capability towards resolving the rare class problem is only a recent discovery [78,79], to be given more applied research attention.

### 3.3. Recognition-Based Approach

This is also known as a one-class learning approach. Some machine learning algorithms including, but not limited to, fuzzy classifiers, decision trees, neural networks, and support vector machines are prone to identifying the majority class occurrences, having been trained to obtain the overall accuracy, to which the rare class contribution is but minimal. A one-class or recognition-based approach therefore offers a solution in which the classifier is modelled on the examples of the non-prevalent class (rare class), not considering the examples from the prevalent class. This approach is particularly useful when instances from the target class are scarce or hard to obtain [38,39]. A recognition-based approach has been reportedly applied in conjunction with autoencoder-based classifiers, neural networks, ensemble classifiers, and SVMs [80,81,82]. Ref. [83] used a fuzzy one-class SVM on imbalanced data to detect fall in a smart room. Likewise, [84,85] reported the successful use of a one-class learning approach in document classification based on SVMs and autoencoders, respectively. Unlike the conventional SVM, a one-class SVM identifies instances from one group instead of differentiating all instances [38]. While considering an imbalanced genomic dataset, [81] showed that one-class SVMs outperform the conventional binary-class SVMs. The study further reported that one-class learning is specifically advantageous when used in a highly dimensional, exceptionally imbalanced, and noisy feature data space. Another important advantage of one-class learning is that it disallows the use of synthetic or fake minority data, which has exposed the results of previously built models to doubts when it comes to a model’s generalization. In the work of [86], a one-class ensemble classifier was tested on 20 different datasets and accuracy results via decision tree and KNN classifiers outperform other resampling methodologies. Notwithstanding the versatility of one-class learning in handling the imbalanced data problem, [39] reported a notable setback as being its inability to handle some machine learning algorithms like the Naïve Bayes, associative classifications, and even decision trees simply because these classifiers were built from samples of more than one class.

### 3.4. Cost-Sensitive Learning

This is employed in practical situations where the misclassification costs are also paramount, not only the data distribution skewness. The majority of the traditional learning algorithms tend to disregard the difference between types of misclassification errors by assuming all misclassification errors cost the same. Cost-sensitive learning methods build on the merit of the fact that it is less expensive to misclassify a true negative occurrence than a true positive occurrence. The methods therefore for a two-class problem assign greater cost to false negatives than to false positives and thereby improve performance with regard to the positive class [39,87]. Research has also attested to combining cost-sensitive learning with oversampling techniques to improve the effectiveness of uplifting model performance [88]. In cost-sensitive learning, cost-sensitive functions are either optimized directly or cost-insensitive algorithms converted to cost-sensitive algorithms by adopting various methodologies of weighting, thresholding, sampling, and ensemble learning [89,90,91,92]. Drawbacks with a cost-sensitive learning approach however includes the assumption that the misclassification costs are known which is rarely the case in real situations. Cost-sensitive classifiers are also known to be prone to data overfitting during training [93], and so extra care must be taken in the calibration stage with this approach.

### 3.5. Ensemble Methods

Ensemble-based methods, also called multiple classifier systems [94,95], are known to merge the performances of many classifiers to produce a single aggregate prediction which outperforms any other classifier considered individually [38,41]. Ensembles of classifiers have been presented as a viable solution to the imbalanced data distribution problem [41,96,97,98,99,100]. The ensemble framework is usually built from a combination of various existing ensemble learning algorithms and any of the earlier discussed approaches including mostly data resampling and cost-sensitive learning. The commonly adopted ensemble learning algorithms are the bagging, boosting, voting, and stacking algorithms [72,96], of which bagging and boosting are the most popular. Bagging, initially developed by [101] works by training individual classifiers using different bootstraps of the dataset. The most widely known bagging algorithm is the random forest [102]. Boosting was proposed by [7,103] to train a sequence of classifiers on difficult learning instances. For an imbalanced data situation, boosting functions by iteratively uplifting classifier performance via updating misclassification cost or by modifying the data distribution ratio [99,104,105]. A detailed classification of the ensemble methods for learning imbalanced data has been extensively described elsewhere [106]. The study by researchers in [106] showed that classifier ensemble-based results outperform results obtained from using data resampling techniques in conjunction with training a single classifier. Simple ensemble approaches like the RUSBoost and UnderBagging have also been reported to outperform many other more complex algorithms [41,107].

### 3.6. Deep Learning Architecture

Deep learning is a seriously advancing field in today’s era of big data and high computing capabilities. It is a versatile algorithm known for automatically extracting attributes from raw data towards regression, classification, and detection analyses. Even though deep learning applications are becoming popular in various fields of endeavor, [10] noted that most of the present solutions do not consider the problem of imbalanced data. A notable major challenge with deep learning architecture is the non-availability of large amounts of data for training and validation [66]. Incorporating this state-of-the-art tool in agri-food applications must appropriately put into consideration the issue of imbalanced data. A recent work by [108] attempted using four deep convolutional neural network (DCNN) models to classify quality grades of cashew kernels. The work entails using an artificial neural network and feature selection to predict breakage rate of maize kernels based on mechanical properties. Likewise, [109] used principal component analysis network (PCANet) in building a predictive model for rice varieties classification. In relation to egg fertility studies, [110] used transfer learning alongside a convolutional neural network (CNN) for classifying 5-day incubated eggs. In the same vein, [111] adopted a multi-feature fusion technique based on transfer learning to classify chicken embryo eggs. In all the cases mentioned above, small datasets were used and addressing imbalanced data was not explicitly considered. Generalization of such built models in real practice is therefore prone to doubts. Knowing that robust models from a deep learning approach must have considered large enough calibrating data, it is glaring that research attempts applying deep learning techniques to agri-food processes and with respect to addressing the imbalanced problem remain at the preliminary stages.

Table 4 shows a summary presentation of the comparison among the described methodologies as grouped into data (resampling, feature selection, one-class learning); algorithm (cost-sensitive and deep learning); and hybrid (ensemble/combination)-driven approaches. The advantages and disadvantages of each of the methods are listed with a view to assisting with making a choice/combination of choices during implementation.

## 4. Evaluation Metrics for Imbalanced Data Analysis

The evaluation metrics adopted are very critical for classification performance assessment and modelling guidance. Overall accuracy (well-known traditionally) has been presented as inappropriate for measuring classifier performance in an imbalanced data situation [39,42], when considered alone. For example, a classifier might obtain 99% overall accuracy in an imbalanced dataset comprising of 99% examples of the prevalent class. This kind of result is misleading and therefore other measures have been proposed for an imbalanced data distribution scenario. Such measures summarizing the performance of a classifier are as shown in a confusion matrix displayed in Table 5 [32]. Other metrics of importance apart from those directly elucidated from the confusion matrix include precision, recall, precision-recall curve, positive and negative predictive value, F-measure, G-mean, receiver operating characteristic (ROC) curve, and area under the curve (AUC).

In a binary class classification situation, the class with very few training samples but with high identification importance is commonly referred to as the positive class and the other as the negative class [5]. This definition, however, seems not to be directly applicable to most agricultural and food processing applications. For example, even though non-fertile eggs in the chicken egg fertility assessment study belong to the rare class (few training data), fertile eggs of the majority class are of higher identification importance, from the hatchery industries’ point of view. Therefore, agricultural and food processing applications might not fit in directly to the definition of the positive class being the class with very few training samples and simultaneously of higher recognition importance. Nonetheless, the definitions of the acronyms in Table 4 remain unchanged with the background understanding of class definitions. These definitions according to [5,32,126] are thus described:

True positive rate (TPR): Proportion of actual positive instances that are predicted as positive:

(TPR = TP/(TP + FN) × 100)(1)

True negative rate (TNR): Proportion of actual negative examples that are predicted as negative:

(TNR = TN/(TN + FP) × 100)(2)

False positive rate (FPR): Proportion of actual negative examples that are predicted as positive:

(FPR = FP/(FP + TN) × 100)(3)

False negative rate (FNR): Proportion of actual positive instances that are predicted as negative:

(FNR = FN/(FN + TP) × 100)(4)

Error rate (ERR) and overall accuracy (OVA) can as well be computed from above as:

ERR = (FP + FN)/(TP + FN + FP + TN) × 100(5)

OVA = (TP + TN)/(TP + FN + FP + TN) × 100 = 1 − ERR(6)

Sensitivity: this is also known as “recall” (R) in information retrieval systems, or “true positive rate” (TPR) as earlier described.

Specificity: this is also known as “true negative rate” (TNR) as earlier described.

Positive Predictive Value (PPV): proportion of predicted positives that are actual positives. This is also called “precision” (P) in information retrieval systems. It must be noted that “precision” might also be described in terms of the negative predictive value in a situation where the rare class has not been taken as the positive class.

PPV = P = TP/(TP + FP) × 100(7)

Negative Predictive Value (NPV): proportion of predicted negatives that are actual negatives (NPV = TN/(TN + FN) × 100). 

F-measure: when only the performance of the positive class is critical, two measures, namely TPR or recall (R) and PPV or precision (P) are adequate. F-measure has been reported as a versatile method for comparing classifier performance in terms of precision to recall, thereby integrating averagely the two measures [127]. F-measure is usually represented as the harmonic mean of precision and recall thus:

(8) $$F-measure=\frac{2PR}{P+R}$$

G-mean: in situations where both performances of the positive and negative classes are paramount, both TPR and TNR are expected to be simultaneously high enough. Hence, [128] proposed the G-mean metric to measure the balanced performance of a classifier between two classes as:

(9) $$G-mean=\sqrt{TPR\times TNR}=\sqrt{Sensitivity\times Specificity}$$

Another version of the G-mean that concentrates majorly on the positive class has been presented below by exchanging the specificity term with the precision term as [129]:

(10) $$G-mean2=\sqrt{TPR\times PPV}=\sqrt{Sensitivity\times Precision}$$

### 4.1. ROC Analysis

**Figure 1:**
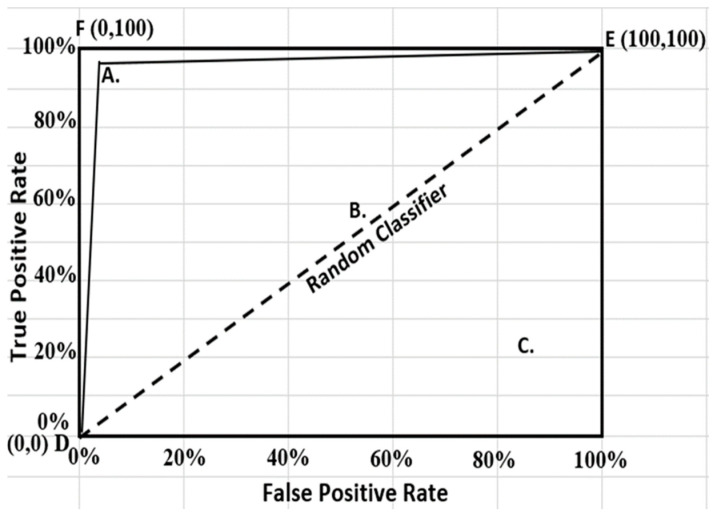
Receiver operating characteristic (ROC) curves for different classifiers: A—good model, B and C—poor models.

ROC graphs have long been in existence and widely used in the field of signal theory and detection [130,131]. It has been equally extended for use in visualizing and analyzing behavior of diagnostic systems [132], and it is a well-known tool in dynamic medical and biomedical research [133]. The earliest use of ROC in machine learning was however traced to the work of [134], who evidently revealed the capability of ROC curves in evaluating and comparing algorithms. The machine learning community in recent times has witnessed an increase in the use of ROC charts partly due to the understanding that the conventional overall accuracy approach is a substandard yardstick for performance evaluation [135,136]. ROC graphs have been shown to be specifically useful in the skewed class distribution and unequal classification error costs domains. These attributes have made ROC analysis increasingly important especially in the present emerging fields of cost-sensitive and imbalanced data learning [137]. ROC analysis examines the interrelationship between sensitivity (TPR) and specificity (TNR) of a binary classifier. Due to prediction changes from score threshold variation, measurements in pairs (FPR, TPR) are generated for each selected singular threshold value [5]. These measurements are connected in a receiver operating characteristic (ROC) curve, having the true positive rate (TPR) on the Y-axis and the false positive rate (FPR), usually denoted as one minus true negative rate (1-specificity), on the X-axis (Figure 1). The optimal classifier “F”, the ideal or perfect model, is that which achieves a false positive rate of 0% but sensitivity or true positive rate of 100% (FPR = 0, TPR = 100). Hence, a good classification model is usually positioned as close as possible to the upper left corner of the graph such as model “A”, while a model making a random guess would be located along the main diagonal (DBE), connecting the points (TPR = 0, FPR = 0) and (TPR = 100, FPR = 100). Therefore, any model positioned on the diagonal such as model “B”, or below the diagonal like model “C”, are considered poor. ROC is thereby shown to depict relative trade-offs between costs (false positives) and gains (true positives). Further description of ROC curves and their implementation can be obtained from [41,138,139,140].

### 4.2. Area Under ROC Curve (AUC)

Since ROC curves show a two-dimensional representation of classifier performance, there is usually a need during classifier comparison analysis to reduce ROC performance to a single scalar value depicting the expected performance [137]. AUC gives such a singular measure of a classifier’s performance for investigating which model is preferable on average [41,141]. AUC, being a portion of the area of the 100%-unit square, will always have values between 0 and 100%. However, having the random guessing positions on the diagonal line between points (0,0) and (100,100) with an area of 50% or 0.5, there cannot be any good classifier with an AUC that is less than 50% [122]. Ref. [142] in a metabolomic biomarker discovery study assessed the utility of model features based on AUC values (%) as follows: 90–100 = excellent; 80–90 = good; 70–80 = fair; 60–70 = poor; and 50–60 = fail.

### 4.3. Precision-Recall (PR) Curve

**Figure 2:**
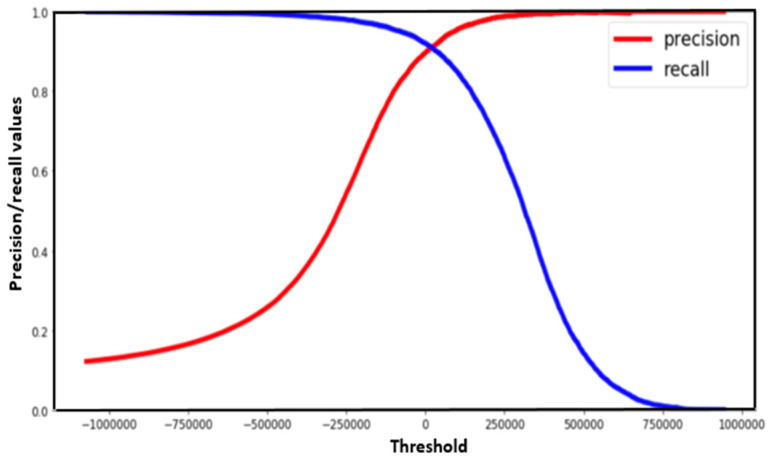
Typical precision-recall curve for best threshold identification.

**Figure 3:**
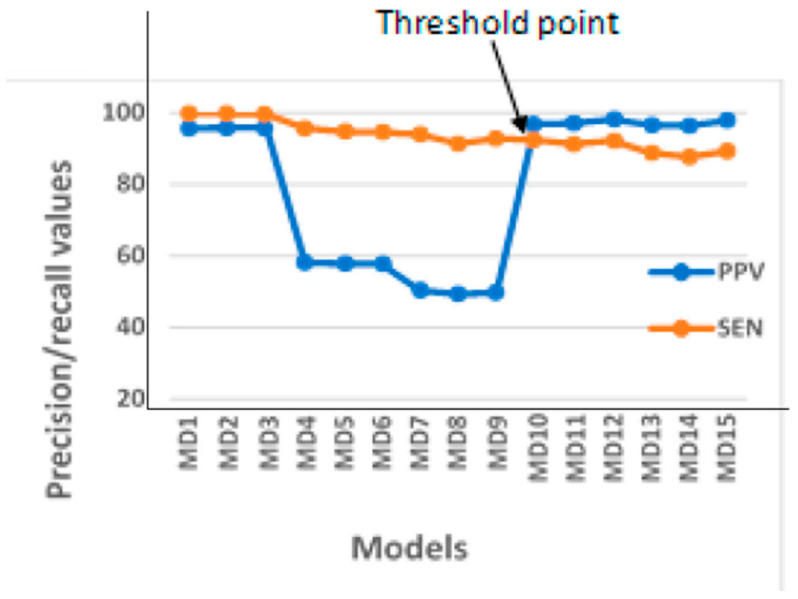
Typical precision-recall curve for optimal model identification (PPV-positive predictive value (precision), SEN- sensitivity (recall), MD1-MD15: Model1-Model15).

There exists situations where there is a need for both precision and recall being high enough and hence necessitating the determination of a safe threshold value for this determination. The trade-off between precision and recall in such situations can be easily observed using the PR curve as depicted in Figure 2 and Figure 3 [143,144]. Figure 3 shows a specific case of the chicken egg fertility classification situation, in which the best threshold point was obtained in identifying the optimal model using the PR curve. 

### 4.4. Comparisons of Evaluation Metrics for Handling Imbalanced Data

With a view to understanding in brevity the various metrics’ advantages and disadvantages in evaluating algorithm performances in an imbalanced scenario, Table 6 shows a comparison summary presentation of the discussed evaluation metrics in terms of the ranking (ROC, AUC, cost, and precision-recall curves) and the threshold (accuracy and F-measure/G-mean) metrics. The threshold metrics with a multi-class focus (including accuracy: error rate, Cohen’s and Fleiss’ kappa measures) consider the overall performance of the learning algorithm and so do not perform optimally in an imbalanced scenario [42,129]. The comparison hereby initially enunciated is based on the single-class focus metrics including accuracy: precision/recall, true positive/true negative rates (sensitivity/specificity), geometric mean (G-mean), and F-measure. 

Other metric measures for imbalanced learning including, but not limited to, ranking (H-measure, AU PREC, B-ROC) and threshold (macro-averaged accuracy-MAA, mean-class-weighted accuracy-MCWA, optimized precision, adjusted geometric mean-AGm, index of balanced accuracy-IBA) metrics have been discussed elsewhere [129,145]. The H-measure tends to alleviate criticism of AUC in relation to having different classifier weightings in misclassifying instances. There are however countering reports that such criticism cannot be generalized and that more practical experiments are needed to demonstrate the superiority of the H-measure over AUC [146,147]. Area under PR curve (AU PREC) is to PR curves what AUC is to ROC, and it has been reported to be more adaptive to changing class skewness than the AUC [129]. Another improvement on ROC analysis is the Bayesian ROC (B-ROC), having the following advantages in handling highly imbalanced datasets: (1) true positive rates are plotted against precision instead of against false positive rates, allowing user control for a low false positive rate; (2) unlike ROC analysis, B-ROC allows graphing of different curves for different class skewness; and (3) unlike in ROC where both misclassification costs and class skewness must be known for classifier comparisons, B-ROC totally avoids the use of estimated misclassification cost in the difficult circumstances of unknown misclassification costs [148].

The macro-averaged accuracy (MAA) is usually computed as the arithmetic mean of the partial accuracies of each class and is presented mathematically as:

MAA = ^1^/_2_ × (Sensitivity + Specificity) (11)

Mean-class-weighted-accuracy (MCWA) is an improvement over MAA earlier introduced by [60]. MCWA is simply MAA with an additional user-defined weight component, thereby able to handle the situation where sensitivity and specificity are not of equal weighting. MCWA is usually presented mathematically as:

MCWA = w × Sensitivity + (1 − w) × Specificity(12)

where ‘w’ is the positive class-assigned weight with values ranging from 0 to 1.

Optimized precision has the main goal of optimizing both specificity and sensitivity rather than weighing contribution of classes according to some domain requirements. The mathematical formula for computing optimized precision has been presented by [149] as:

(13) $$Optimized\;Precision=Specificity\;\times \;N_{n}\;+\;Sensitivity\;\times \;N_{p}\;-\;\frac{\left|Specificity\;-\;Sensitivity\right|}{Specificity\;+\;Sensitivity}$$

where ‘Nn’ and ‘Np’ stand for the number of negative and positive instances, respectively, in the dataset.

The adjusted geometric mean (AGm) was introduced to tackle the imbalanced problem in situations of increasing sensitivity at the expense of specificity. Even though the F-measure tends to address this problem via parameter manipulation, identifying the right parameter value of use remains a challenge when the misclassification costs are not specifically known. The AGm as proposed by [150] is computed as:

(14) $$AGm\;=\;\frac{G\;-\;mean\;+\;Specificity\;\times \;Nn}{1\;+\;Nn},\;if\;Specificity\;>\;0,\;AGm\;=\;0,\;if\;sensitivity\;=\;0$$

The index of balanced accuracy (IBA) shows that the various combinations of specificity and sensitivity can produce the same G-mean values and therefore proposes a generalized IBA mathematical formulation as:

IBAα (M) = 1 + α × Dom) × M(15)

where Dom (dominance) is defined as sensitivity – specificity, M is any metric, and α is a weighting factor introduced to reduce dominance influence on metric M’s result. IBAα from theoretical and experimental analyses has been shown to have high correlation with G-mean and AUC, which makes it a versatile metric tool in handling class-imbalanced datasets.

## 5. Conclusions and Future Research Direction

This review has presented the reality of the imbalanced data problem in agri-food applications, with demonstration of the criticality of addressing the problem using examples from non-agri-food and agri-food data analysis scenarios. Appropriate multivariate downstream analysis is becoming a very serious issue of concern in the food processing sector due to non-robustness of available models built from imbalanced data. This paper therefore discussed some of the existing approaches that have been found useful in tackling the imbalanced problem in other fields of endeavor like in the medical, information technology, and production engineering fields. Such approaches, including, but not limited to, resampling, feature extraction/selection techniques, one-class learning, cost-sensitive learning, ensemble methods, and deep learning architectures were examined for adoption consideration in the agri-food sector. Deep learning without doubt is gaining wide popularity in the present big data era; it was however observed that most of the common solutions do not consider the problem of data skewness, and its appropriate implementation in agri-food applications with respect to addressing the imbalanced problem remains preliminary and must therefore be given more in-depth attention.

Despite enough available evidence of obtaining substantive model upliftment when data imbalance was addressed during analysis of non-agri-food data, there have not been rigorous research efforts in the direction of using existing state-of-the-art approaches for solving data imbalance problems in the agri-food data analysis context. This review therefore is a forward step taken towards sensitizing agri-food researchers to concentrate more in this area of research. There will be need for increased experimental study subjecting imbalanced agri-food data to appropriate analysis approaches as discussed in this paper. Future research directions along the line of the typical case of chicken egg fertility classification, discussed here using a 10-fold cross-validated sampling approach, will entail testing built predictive models using real unknown data that have not been captured in the calibration/training stage of modelling. If synthetic samples have been used for resampling as is common with the SMOTE method, final verification of the model should be tested with unknown samples excluding the synthetic instances. This kind of testing results will showcase the closest expected outcome when such a model is deployed in practice.

The comparative analysis of methods and metrics were helpful in understanding similarities and differences and therefore being able to examine clearly the advantages and disadvantages of using any or a combination of the methods and metrics. In the future, this study will consider the feasibility of building optimal robust models using the discussed methods and metrics, in conjunction with assessing performance of some combination of classifiers. 

Rightly analyzing food data and using appropriate performance evaluation metrics can improve accuracy of results and model robustness. This has great potential to further enhance the acceptability and adoptability of innovations/inventions in agri-food predictive modelling research efforts. It is believed that the right implementation of the outcome of this paper would find useful applications in appropriate handling of agri-food imbalanced data during multivariate downstream analysis.

## Figures and Tables

**Table 1:** Typical dataset characteristics in the computer sciences field (network intrusion detection).

Data	Majority	Minority	Ratio	ZeroR Acc	Ref.
CICIDS2017	2,800,000	14	1:200,000	99.9%	[16]
UNSW-NB15	2,540,044	9	1:282,000	99.9%	[17]
KDD99	4,898,430	4	1:1,200,000	99.9%	[17]
CSE-CIC- IDS2018	16,200,000	6	1:2,700,000	99.9%	[18]

Modified from [11].

**Table 2:** Modeling results prior to (PH) and after (AH) handling imbalanced distribution.

Data	Classifier	Accuracy PH, AH (%)	F1-Score PH, AH (%)	Recall PH, AH (%)	Precision PH, AH (%)	Ref.
CICIDS2017	LightGBM	99.86, **99.91**	-, -	-, -	-, -	[19]
CICIDS2017	AdaBoost	-, **81.83**	-, **90.01**	-, **100.00**	-, **81.83**	[20]
UNSW-NB15	KNN RF	84.00, **95.10** 84.00, **95.10**	53.30, **95.10** 53.30, **95.10**	51.30, **95.70** 51.30, **95.70**	57.80, **94.80** 57.80, **94.80**	[9]
UNSW-NB15	GRU	57.00, **77.90**	71.30, **79.00**	97.3, **83.20**	56.30, **75.30**	[21]
CSE-CIC- IDS2018	KNN Adaboost	98.52, **98.80** 99.69, **99.60**	98.89, **98.00** 99.70, **99.60**	98.52, **98.08** 99.69, **99.61**	99.28, **97.92** 99.70, **99.60**	[22]
KDD99	CNN LSTM	92.30, **95.20** 91.80, **95.40**	95.20, **94.90** 94.70, **95.10**	91.00, **90.70** 91.10, **91.40**	99.80, **99.50** 98.60, **99.40**	[21]

Modified from [11]. Bold values are optimized outputs after tackling the imbalanced situation.

**Table 3:** Typical dataset characteristics in the agri-food sector (chicken egg fertility classification).

Data	Majority	Minority	Ratio	ZeroR	SEN	SPE	AUC	F1-Score
S1	8807	400	1:22	95.70%	99.50%	0.30%	50.90%	49.00%
S1 + S2 (S2)	17,614	800	1:22	95.70%	99.10%	2.90%	62.50%	51.10%
S1 + SMOTE	8807	6400	1:1.4	57.90%	93.10%	97.10%	97.90%	94.70%
S2 + SMOTE	17,614	12,800	1:1.4	57.90%	93.00%	98.10%	98.10%	95.10%
S1 + SMOTE + Ru	6400	6400	1:1	50.00%	90.30%	97.50%	97.50%	93.90%
S2 + SMOTE + Ru	12,800	12,800	1:1	50.00%	90.10%	98.30%	97.50%	94.20%
S1 + SMOTE + RAND	8807	6400	1:1.4	57.90%	93.20%	97.10%	97.90%	94.80%
S2 + SMOTE + RAND	17,614	12,800	1:1.4	57.90%	93.10%	98.10%	98.10%	95.10%
S1 + SMOTE + RAND + Ru	6400	6400	1:1	50.00%	90.10%	97.50%	97.40%	93.80%
S2 + SMOTE + RAND + Ru	12,800	12,800	1:1	50.00%	90.30%	98.30%	97.50%	94.30%

**Table 4:** Comparison of different methods for solving class imbalance.

	Advantages	Disadvantages	Ref.
**Data**	Classifier independent, class instance information preservation	Prone to overfitting due to exact duplication in simple oversampling	[42,73]
	Performance improvement for ensemble classifiers (sampling + clustering + feature selection)	Computational complexity and data-dependency	[112]
	Selection of representative prevalent sample (spatial undersampling)	Non-fitting the entire distribution of the original unclassified data	[113]
	Solving small disjuncts problem in oversampling	Challenge with handling big datasets and solving multi-class imbalance	[114]
	Consideration of selective generation strategies and intra-class data variation (global local-based oversampling)	Model complexity and data dependency	[115]
	Performance improvement via autonomous sample selection (sampling strategies + reinforcement learning)	Data feedback reliance and algorithm complexity	[116]
	Efficient in a highly dimensional and noisy feature data space imbalanced situation (one-class)	Recognition-based learning is algorithm-specific	[39]
	Disallows use of fake and synthetic data (one-class learning)	The learning class must be large enough for algorithm recognition of inherent discriminating features	
**Algorithm**	Versatile in practical situations where misclassification costs are paramount (cost-sensitive)	Prone to overfitting and it is also based on assumption that misclassification costs are known	[39,93]
	Very effective in the presence of big amount of data (deep learning)	Unavailability of large enough training and validation data in practice	[66]
	Immune to high noise and of low computational complexity	Data distribution vulnerability	[117]
	Improved F1-score and AUC in a rare class (cost-sensitive SVM)	Cannot handle multiclass imbalance and big datasets	[118]
	Able to handle dynamic imbalance problem	Model complexity and long run times	[119]
**Hybrid**	Low complexity, simple and efficient (Adaptive Weighted ensemble BLS)	Cannot tackle multi-class imbalanced problem	[120]
	Effective categorization of extreme data imbalance	Complex models and long run times	[121]
	Noise detection ability in imbalanced distribution	Inability and poor adaptability to solving multiclass imbalance	[122]
	Consideration of base classifiers’ diversity	Cannot tackle multiclass imbalance	[123]
	Effective boundary samples and noise resolution	Large amount of run time consumption	[124]

Modified from [125].

**Table 5:** Typical dataset characteristics in the agri-food sector.

		Prediction Class	
		Predicted as Positive	Predicted as Negative
**True Class**	**Actual Positive**	TPR True Positive Rate	FNR False Negative Rate
	**Actual Negative**	FPR False Positive Rate	TNR True Negative Rate

**Table 6:** Comparison of different evaluation metrics for solving class imbalance.

	Advantages	Disadvantages
**Ranking**	Performance in each category is broken down into two separate measures with a multi-class interpretability possibility at glance (ROC)	Validity of ROC analysis is dependent on the false and true positive rates being invariant to class skewness. The analysis might not be fully trusted in the case of changing class distributions Precision-recall curve thrives better in such situation
	Can evaluate eventualities in various situations, including when the domain imbalance ratio is not exactly known (ROC)	Readability difficulty in situation of known cost/imbalance ratio
	Good summary statistics singular metric measure (AUC)	Loss of cogent information on algorithm performances over the whole operating range
**Threshold**	Not affected by class imbalance since the correctly classified proportions of the classes are identified separately (sensitivity/specificity)	More difficult to process the metrics as a measure for the combined classes than for each single class
	A great measure for all predicted instances assigned to a given class (sensitivity/specificity)	Misses identifying the proportion of instances assigned to a given class that actually belong to this class
	Precision measure identifies the proportion of predicted instances assigned to a given class (usually positive class), which actually belongs to that class.	Must be considered together with recall for understanding the overall classifier performance on the positive class with no performance information on the negative class
	Single metric considering classifier performance on both the positive and the negative classes (G-mean)	Usage limited to classes assumed to be of equal importance
	Single metric for classifier performance from precision and recall values (F-measure)	Applicable to a single class (usually the positive class) per time

Modified from [129].

## Data Availability

The original contributions presented in the study are included in the article, further inquiries can be directed to the corresponding authors.
